# Constrained Soft Actor–Critic for Joint Computation Offloading and Resource Allocation in UAV-Assisted Edge Computing

**DOI:** 10.3390/s26041149

**Published:** 2026-02-10

**Authors:** Nawazish Muhammad Alvi, Waqas Muhammad Alvi, Xiaolong Zhou, Jun Li, Yifei Wei

**Affiliations:** Beijing Key Laboratory of Work Safety Intelligent Monitoring, School of Electronic Engineering, Beijing University of Posts and Telecommunications, Xitucheng Road No. 10, Beijing 100876, China; 2024090003@bupt.cn (W.M.A.); 2024040009@bupt.cn (X.Z.); lijun2021@bupt.edu.cn (J.L.); weiyifei@bupt.edu.cn (Y.W.)

**Keywords:** UAV-assisted edge computing, computation offloading, constrained reinforcement learning, Markov decision process, latency constraints, resource allocation

## Abstract

Unmanned Aerial Vehicle (UAV)-assisted edge computing supports latency-sensitive applications by offloading computational tasks to ground-based servers. However, determining optimal resource allocation under strict latency constraints and stochastic channel conditions remains challenging. This paper addresses the joint computation partitioning and power allocation problem for UAV-assisted edge computing systems. We formulate the problem as a Constrained Markov Decision Process (CMDP) that explicitly models latency constraints, rather than relying on implicit reward shaping. To solve this CMDP, we propose Constrained Soft Actor–Critic (C-SAC), a deep reinforcement learning algorithm that combines maximum-entropy policy optimization with Lagrangian dual methods. C-SAC employs a dedicated constraint critic network to estimate long-term constraint violations and an adaptive Lagrange multiplier that automatically balances energy efficiency against latency satisfaction without manual tuning. Extensive experiments demonstrate that C-SAC achieves an 18.9% constraint violation rate. This represents a 60.6-percentage-point improvement compared to unconstrained Soft Actor–Critic, with 79.5%, and a 22.4-percentage-point improvement over deterministic TD3-Lagrangian, achieving 41.3%. The learned policies exhibit strong channel-adaptive behavior with a correlation coefficient of −0.894 between the local computation ratio and channel quality, despite the absence of explicit channel modeling in the reward function. Ablation studies confirm that both adaptive mechanisms are essential, while sensitivity analyses show that C-SAC maintains robust performance with violation rates varying by less than 2 percentage points even as channel variability triples. These results establish constrained reinforcement learning as an effective approach for reliable UAV edge computing under stringent quality-of-service requirements.

## 1. Introduction

The advancement of sixth-generation (6G) wireless networks is driving unique demands for intelligent, adaptive systems capable of supporting latency-sensitive applications such as autonomous vehicles, industrial automation, and extended reality. Unmanned Aerial Vehicles (UAVs) have emerged as a key enabler for such applications by offering deployment flexibility, favorable line-of-sight channels, and the capability to provide on-demand coverage where terrestrial infrastructure is lacking [1]. When UAVs are equipped with sensing and computing capabilities, they can serve as mobile edge nodes by acquiring environmental data and performing real-time processing for critical decision-making [2]. One of the major challenges associated with UAV-assisted edge computing is determining how to split computational tasks between processing them locally on the UAV’s resources and transmitting them to ground-based edge servers for processing [3]. Local processing eliminates transmission delays, but it limits the processing capability due to limited availability of onboard resources. On the other hand, offloading allows the use of superior processing capabilities at the servers but it depends on the quality of the wireless channel and consumes significant transmission energy [4]. Determining how to split the tasks is particularly difficult when there are strict latency requirements, since missing deadlines can lead to serious consequences, ranging from degraded user experience to safety-critical failures [5]. Furthermore, the stochastic nature of wireless channels causes offloading performance to vary randomly [6]. In general, optimizing this process using traditional optimization techniques relies on accurate mathematical models. These techniques typically assume that perfect channel state information (CSI) is available. This is impractical for many real-world deployments, where the environment changes randomly and over time [7]. Recently, deep reinforcement learning (DRL) has been identified as a promising alternative method for such problems. DRL enables model-free policy learning that adapts to complex and stochastic environments without requiring explicit knowledge about the system [8]. There has also been significant interest in recent years in applying DRL to optimize the computation offloading process in edge computing systems [9,10]. However, many DRL methods rely on either implicit or explicit reward shaping to solve these problems. DRL techniques also rely on penalty terms to enforce the latency constraints. This may require large amounts of hyperparameter tuning and provides little guarantee of satisfying the constraints. This is particularly problematic in applications where meeting deadlines is absolutely necessary. Constrained reinforcement learning is a framework that uses Lagrangian relaxation as a means of solving optimization problems subject to constraints. Although this approach has been extensively adopted in prior studies for wireless resource allocation [11,12], existing works have not considered UAV-assisted edge computing scenarios with continuous computation partitioning. As a result, a clear disconnect remains between recent advances in constrained learning methods and their practical deployment in UAV-enabled edge computing systems. In this study, we bridge this gap by developing a Constrained Soft Actor–Critic (C-SAC) algorithm for UAV-assisted edge computing with latency constraints. We first formulate the joint computation partitioning and power allocation problem as a Constrained Markov Decision Process (CMDP), and then employ Lagrangian dual optimization to handle the constraints in a principled manner. Our approach builds upon the maximum-entropy framework [13] and utilizes stochastic policies, which provide inherent robustness to uncertainty in the wireless channel. This property does not hold for deterministic methods, which are the most common approaches currently employed in the literature [14]. Our research contributes to the literature in the following ways:

1. Unlike prior works that rely on binary or partial offloading with heuristic optimization for split transmission and joint resource allocation (e.g., game-theoretic methods [15] or convex approximations [16]), we formulate the joint computation partitioning and power allocation problem for UAV-assisted edge computing as a CMDP with continuous action spaces [17]. This formulation explicitly captures the coupling between offloading decisions and wireless resource allocation under hard latency constraints in a single stochastic model, without relying on implicit penalties or deterministic simplifications. Our approach extends traditional optimization methods [18] and unconstrained RL techniques [19,20] by incorporating stochastic policies to better handle stochastic channel dynamics and strict QoS requirements in high-variance UAV-MEC environments.
2. We propose a constrained reinforcement learning algorithm called C-SAC, which combines maximum-entropy policy optimization with Lagrangian dual methods. C-SAC is particularly well-suited for UAV-assisted edge computing because (i) its stochastic policies provide inherent robustness to the high-variance wireless channels characteristic of air-to-ground links, where deterministic policies fail under rapid channel fluctuations [21]; (ii) the maximum-entropy framework encourages exploration across the continuous action space of partitioning ratios and power levels, critical for discovering energy-efficient solutions in the complex coupling between computation and communication decisions; (iii) it proactively avoids long-term latency issues rather than just fixing immediate ones, which is essential for applications where latency is critical for safety [22]; and (iv) the adaptive Lagrange multiplier automatically balances energy efficiency against latency satisfaction without manual hyperparameter tuning, addressing the impracticality of manual tuning in dynamic UAV deployments. This makes C-SAC superior to deterministic methods like TD3-Lagrangian, which exhibit instability under channel uncertainty, and unconstrained methods like vanilla SAC, which cannot guarantee constraint satisfaction.
3. Extensive experiments show that C-SAC achieves an 18.9% constraint violation rate, representing a 76% relative reduction compared to unconstrained SAC and a 54% relative improvement over deterministic TD3-Lagrangian. The learned policies also exhibit strong channel-adaptive behavior (Pearson correlation of −0.894 between the local computation ratio and channel quality) without explicit channel modeling in the reward function.
4. Finally, we conducted a series of ablation studies and sensitivity analyses demonstrating that C-SAC maintains its performance across a wide variety of channel conditions, with the variation rate being less than 2 percentage points even as the channel variability increases threefold.

The remainder of this paper is organized as follows: Section 2 reviews prior work on UAV-assisted edge computing and constrained reinforcement learning. Section 3 presents the system model, including the task generation model, wireless channel model, computation model, and energy consumption model. In Section 4, we formulate the resource allocation problem as a CMDP. In Section 5, we describe the proposed C-SAC algorithm and baselines. In Section 6, we report the experimental results, ablation studies, and sensitivity analyses. Finally, in Section 7, we conclude the paper and outline several possible avenues for future work.

## 2. Related Work

This section provides a summary of prior related work, focusing on UAV-MEC architectures, task offloading strategies, and constrained reinforcement learning methods to ensure quality of service (QoS) for latency-sensitive tasks in real-time applications.

UAVs are becoming increasingly significant in modern wireless networks as they provide on-demand wireless access and typically a line-of-sight (LoS) connection to end devices [21,23]. The capabilities of UAVs in wireless networks were described in detail by Wu et al. [1], who emphasized their potential as mobile base stations and edge servers. Recently, there have been several studies focused on more dynamic environments; for example, Wang et al. [21] studied both trajectory planning and resource allocation for multiple UAV systems, highlighting the difficulty of coordinating interference and energy consumption. Energy-efficient frameworks for joint trajectory and power optimization were presented by Li et al. [18]. Their approach focused on minimizing the long-term energy usage of the UAVs, rather than meeting strict per-task latency deadlines.

Binary offloading is no longer sufficient to meet the demands of modern MEC. Saeedi et al. [3,24] provided a comprehensive overview of recent advances in offloading techniques, pointing out that continuous task partitioning provides a much finer level of granularity when it comes to resource allocation. Vehicular networks present additional challenges to offloading techniques. The adaptive scheduling method proposed by Ning et al. [25] was shown to increase throughput by balancing workloads between local devices and edge servers. Furthermore, the integration of sensing and communication has created new dimensions to consider when it comes to offloading. Truong et al. [26] examined the use of Reconfigurable Intelligent Surfaces (RISs) to enhance UAV-MEC performance, demonstrating how environmental control can decrease transmission delays in challenging propagation conditions.

More recently, split transmission and partial offloading strategies have been explored extensively using classical optimization techniques for joint resource allocation. Sun et al. [27] addressed multi-UAV-assisted MEC systems by formulating a multi-objective optimization problem that jointly considers task offloading, computation resource allocation, and UAV trajectory control. Their approach employs block alternate descent combined with distributed splitting, threshold rounding, Karush–Kuhn–Tucker (KKT) conditions, and successive convex approximation (SCA) to minimize task completion delay and UAV energy consumption while maximizing the number of offloaded tasks. Similarly, Qin et al. [28] investigated RIS-assisted UAV-enabled MEC systems with non-orthogonal multiple access (NOMA), proposing an iterative algorithm based on Dinkelbach’s method and block coordinate descent (BCD) to jointly optimize bit allocation, transmit power, phase shift, and UAV trajectory for energy efficiency maximization. Their work demonstrates that Lagrangian dual methods and DC programming can effectively handle the coupled optimization variables in UAV-MEC environments. Additionally, Yong et al. [29] proposed an alternating optimization algorithm (EPSO-GA) for partial subtask offloading in D2D-enabled MEC networks, achieving low latency and energy costs through joint optimization of transmit power allocation and subtask offloading strategies. Building on earlier non-ML approaches such as game-theoretic methods [15] and convex approximations [16,18], these optimization-based works provide fine-grained control over offloading decisions. However, they generally assume known and stationary system models with perfect or near-perfect channel state information (CSI), making them less suitable for the stochastic and time-varying channel conditions characteristic of UAV air-to-ground links. This limitation motivates the use of model-free reinforcement learning approaches that can learn adaptive policies directly from interaction with the environment.

DRL has proven to be a valuable tool for addressing the randomness inherent in wireless channels. Luong et al. [30] pointed out that while many previous methods used value-based approaches, actor–critic methods are generally preferred for continuous action space problems, such as power and resource allocation. Another study by Kong et al. [20] employed a TD3 algorithm to investigate task offloading in maritime edge computing, achieving excellent results. However, as previously mentioned, most DRL-based methods, including those by Wang et al. [19,31], rely on reward shaping to satisfy constraints. Reward shaping is effective in terms of average performance; however, it does not guarantee the satisfaction of strict latency requirements, as the agent may still prefer rewards over satisfying the constraints in edge cases.

To address the limitations of standard DRL, constrained reinforcement learning (CRL) has attracted considerable attention [32]. By modeling safety and latency requirements as Constrained Markov Decision Processes (CMDPs), researchers can incorporate them directly into the problem formulation. Paternain et al. [32] developed the theoretical foundation of the primal–dual methods in CMDPs, demonstrating their convergence in complex environments. In wireless applications, Liu et al. [33] applied CRL to network slicing, demonstrating that Lagrangian-based methods can maintain the reliability of latency requirements far better than unconstrained baselines. In recent years, Fasihi et al. [34] proposed state-augmented algorithms, which further improved the reliability of satisfying the constraints in highly dynamic 6G environments.

Although the literature contains many improvements in the field, there exists a significant research gap concerning the combination of continuous computation partitioning with explicit, hard latency constraints in UAV-assisted systems [23,24,34]. As shown in Table 1, many previous works—including recent optimization-based approaches such as [27,28,29]—treated constraints implicitly through penalty terms or weighted objectives, or used deterministic policies that may not be robust under extreme channel variability. The purpose of this paper is to fill this gap by proposing C-SAC, a stochastic-policy-based method using maximum-entropy optimization combined with an adaptive Lagrange multiplier. Unlike prior optimization-based methods that assume perfect CSI and stationary channels, our approach learns adaptive policies that explicitly satisfy hard latency constraints even under high-variance UAV air-to-ground channel conditions.

## 3. System Model

**Figure 1:**
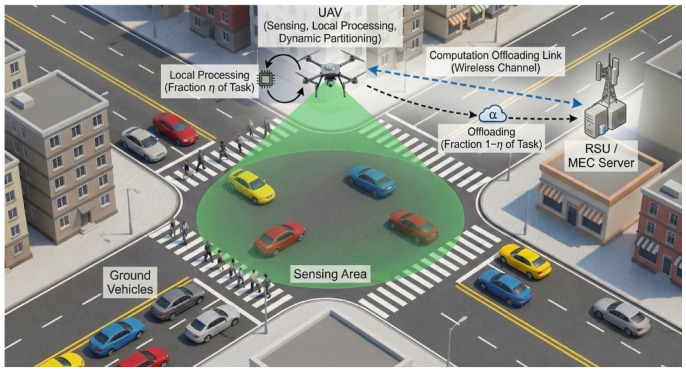
System architecture showing the UAV hovering at altitude *h*, with wireless connectivity to the RSU for computation offloading.

We consider a mobile edge computing system using a UAV for latency-sensitive tasks in 5G and beyond-5G wireless networks. We illustrate this system architecture in Figure 1, where a rotary-wing UAV hovers at a fixed altitude *h* over a roadside intersection and receives continuous data from various sensors for traffic monitoring, incident detection, or autonomous vehicle navigation support. The UAV is connected to a roadside unit (RSU) on the ground equipped with an edge computing server allowing the UAV to offload computation for each task depending on the channel quality and the computational requirements [35]. A critical issue arises when deciding whether to process each UAV task locally or remotely on the server. If processing occurs locally, then there is no transmission delay; however, it consumes more of the UAV’s computing capability than if the UAV were able to offload some of the processing to the server. However, if the UAV decides to offload some or all of its processing to the server, the performance depends on the quality of the communication link between the UAV and the server.

To make this decision in real time while meeting the application and UAV constraints, the decision-maker needs to know the communication link quality and the processing required to complete each UAV task within the maximum allowable delay $T_{\max}$, a common constraint in intelligent transportation systems. Table 2 summarizes the symbols used in this study.

### 3.1. Task Generation

The amount of data generated for each sensing cycle is modeled as a truncated Gaussian random variable with parameters ($\mu_{L}$, $\sigma_{L}^{2}$):

(1) $$L_{t}∼N\left(\mu_{L},\sigma_{L}^{2}\right),\;L_{t}\in \left[L_{\min},L_{\max}\right]$$

The processing required for each sensing cycle is represented as a function of the generated data, i.e., $\kappa L_{t}$ CPU cycles, where κ represents the processing intensity (number of CPU cycles/number of data bits) [36].

### 3.2. Wireless Channel

The UAV communicates with the RSU via a radio frequency link between the ground and the UAV, which uses bandwidth *B*. In this problem, the UAV’s communication channel is modeled using a block fading process, where the UAV experiences a static channel during every time slot, but each time slot has its own independent channel characteristics [21]. Therefore, let $\Gamma_{t}$ represent the UAV’s signal-to-noise ratio (SNR) in decibels at time slot *t*, while transmitting at maximum power $P_{\max}$; then the distribution of $\Gamma_{t}$ can be expressed as a normal distribution [26]:

(2) $$\Gamma_{t}∼N\left(\bar{\Gamma },\sigma_{\Gamma }^{2}\right),\;\mathrm{and}\;\Gamma_{t}\in \left[\Gamma_{\min},\Gamma_{\max}\right]$$

where $\bar{\Gamma }$ is the mean SNR experienced by the UAV when transmitting at the fixed altitude, and $\sigma_{\Gamma }$ represents the variability of the SNR due to other environmental conditions. The achievable data rate $R_{t}$ of the UAV at time *t* is given by the expression

(3) $$R_{t}=B\log_{2}\left(1+10^{\Gamma_{t}/10}\cdot \frac{p_{t}}{P_{\max}}\right)$$

Equation (3) shows how both the channel quality $\Gamma_{t}$ and the transmit power $p_{t}$ impact the effective transmission rate through the use of adaptive resource allocation techniques [37]. Specifically, increasing the transmit power $p_{t}$ directly increases the data rate $R_{t}$, which in turn reduces the transmission time $T_{\mathrm{tx},t}$ for offloaded data (5). However, transmit power does not affect the local computation time $T_{\mathrm{local},t}$ or the edge server computation time $T_{\mathrm{edge},t}$, as these depend solely on the computation partitioning ratio and processor frequencies. Therefore, higher transmit power is particularly beneficial when the UAV offloads a large portion of the task (i.e., when $\eta_{t}$ is low), as it accelerates the offloading path without impacting the local processing path.

### 3.3. Computation Partitioning

For each task with size $L_{t}$, the UAV chooses a split ratio $\eta_{t}\in \left[0,1\right]$, which defines how many of the $L_{t}$ bits should be computed locally. By using a continuous split ratio instead of a discrete (binary) offloading scheme, we can make fine-grained adaptations depending on the current conditions. The time required for computing $\eta_{t}L_{t}$ bits locally on the UAV’s onboard processor operating at frequency $f_{\mathrm{UAV}}$ is given by

(4) $$T_{\mathrm{local},t}=\frac{\eta_{t}L_{t}\kappa }{f_{\mathrm{UAV}}}$$

The remaining $\left(1-\eta_{t}\right)L_{t}$ bits are sent to the edge server for remote processing. The transmission time depends on the current data rate:

(5) $$T_{\mathrm{tx},t}=\frac{\left(1-\eta_{t}\right)L_{t}}{R_{t}}$$

The edge server operating at frequency $f_{\mathrm{RSU}}\gg f_{\mathrm{UAV}}$ computes the offloaded part in

(6) $$T_{\mathrm{edge},t}=\frac{\left(1-\eta_{t}\right)L_{t}\kappa }{f_{\mathrm{RSU}}}$$

Therefore, $\eta_{t}=1$ corresponds to local processing, $\eta_{t}=0$ corresponds to full offloading, and all other values correspond to hybrid execution where the UAV uses both resources to compute the task.

### 3.4. End-to-End Latency

The total time $D_{t}$ needed to complete task *t* is defined as the sum of a fixed sensing duration $T_{\mathrm{sense}}$ and the processing delay. As local computation and offloading use different resources (CPU vs. radio), they can run concurrently. Therefore, the end-to-end delay is determined by the maximum of the two paths:

(7) $$D_{t}=T_{\mathrm{sense}}+\max\left(T_{\mathrm{local},t},\;T_{\mathrm{tx},t}+T_{\mathrm{edge},t}\right)$$

Using the previous equations, we obtain the expanded version of the total delay:

(8) $$D_{t}=T_{\mathrm{sense}}+\max\left(\frac{\eta_{t}L_{t}\kappa }{f_{\mathrm{UAV}}},\;\frac{\left(1-\eta_{t}\right)L_{t}}{R_{t}}+\frac{\left(1-\eta_{t}\right)L_{t}\kappa }{f_{\mathrm{RSU}}}\right)$$

Thus, there is a basic trade-off here: Increasing $\eta_{t}$ results in lower communication costs, but increases the time required to process the task locally. More specifically, increasing $\eta_{t}$ has the following effects on the end-to-end latency $D_{t}$: (i) the local computation time $T_{\mathrm{local},t}$ increases proportionally with $\eta_{t}$ as shown in (4); (ii) the transmission time $T_{\mathrm{tx},t}$ decreases because fewer bits $\left(1-\eta_{t}\right)L_{t}$ need to be offloaded (5); and (iii) the edge computation time $T_{\mathrm{edge},t}$ also decreases for the same reason (6). Since the end-to-end latency is determined by the maximum of the local path and the offloading path (7), the optimal $\eta_{t}$ balances these two competing paths. When channel conditions are poor (low $\Gamma_{t}$), the transmission time dominates, favoring higher $\eta_{t}$ (more local processing). When channel conditions are good (high $\Gamma_{t}$), offloading becomes faster, favoring lower $\eta_{t}$. The best value of $\eta_{t}$ strongly depends on the current channel state $\Gamma_{t}$ and the size of the task $L_{t}$, which motivates an adaptive, learning-based method to find the best partitioning of the task. In addition, the system must meet the constraint on the maximum allowed delay:

(9) $$D_{t}\leq T_{\max}$$

with $T_{\max}=35$ ms selected as a stringent latency constraint for our experimental simulations in this UAV-assisted MEC environment [22].

### 3.5. Energy Consumption

Due to limited battery life, energy efficiency is crucial for UAV-based computations. As shown below, the total controllable energy consumed by the UAV during one time slot consists of three parts:

Sensing Energy: The sensor consumes a constant power $P_{\mathrm{sense}}$ for the whole duration of the sensing operation:

(10) $$E_{\mathrm{sense}}=P_{\mathrm{sense}}\cdot T_{\mathrm{sense}}$$

Computation Energy: The computation energy is proportional to the third power of the clock frequency according to the cubic relationship between the switching power and the clock frequency for CMOS circuits [38]:

(11) $$E_{\mathrm{comp},t}=\zeta f_{\mathrm{UAV}}^{2}\cdot \eta_{t}L_{t}\kappa$$

where ζ represents the effective switched-capacitance coefficient. This parameter is a hardware-specific constant that reflects the energy consumed per CPU cycle, with the value $\zeta =10^{-28}$ J/cycle used as a standard representation for high-performance mobile processors in the MEC literature [39].

Communication Energy: The energy consumed by transmitting the offloaded data is

(12) $$E_{\mathrm{comm},t}=p_{t}\cdot T_{\mathrm{tx},t}=p_{t}\cdot \frac{\left(1-\eta_{t}\right)L_{t}}{R_{t}}$$

The total controllable energy is

(13) $$E_{\mathrm{total},t}=E_{\mathrm{sense}}+E_{\mathrm{comp},t}+E_{\mathrm{comm},t}$$

The computation partitioning ratio $\eta_{t}$ affects the total UAV energy consumption $E_{\mathrm{total},t}$ through two opposing mechanisms: (i) increasing $\eta_{t}$ raises the local computation energy $E_{\mathrm{comp},t}$ proportionally (11), as more bits are processed by the UAV’s onboard processor; and (ii) increasing $\eta_{t}$ reduces the communication energy $E_{\mathrm{comm},t}$ (12), since fewer bits need to be transmitted to the edge server. The transmit power $p_{t}$ directly increases $E_{\mathrm{comm},t}$; however, higher $p_{t}$ also increases the data rate $R_{t}$ (3), which reduces the transmission time $T_{\mathrm{tx},t}$, partially offsetting the energy increase. It is important to note that all energy quantities in $E_{\mathrm{total},t}$ refer to the UAV-side controllable energy expenditure; the edge server’s computation energy is not included in the optimization objective, as it is powered by the grid infrastructure and does not affect UAV mission endurance.

To focus on the impact of computation partitioning and transmit power allocation, we model sensing duration $T_{\mathrm{sense}}$ and sensing power $P_{\mathrm{sense}}$ as fixed constants (common in UAV-MEC offloading studies where sensing is periodic and independent of offloading decisions) [18]. Similarly, under the fixed-altitude hovering assumption, propulsion energy is treated as constant and thus omitted from the optimization objective, as widely adopted in hovering UAV-MEC scenarios to isolate controllable communication and computation energy [40]. While propulsion energy is often the dominant energy component [41], for a rotary-wing UAV in a fixed-altitude hovering state, the propulsion power is a constant offset ($P_{hover}$) and does not affect the optimal resource allocation policy.

### 3.6. Optimization Problem

The main goal of this work is to minimize the total energy consumption while fulfilling the delay constraint. At every time slot, the UAV has to choose two decision variables: the computation partitioning ratio $\eta_{t}$ and the transmit power $p_{t}$. Both decisions are based on the current channel state $\Gamma_{t}$, the size of the task $L_{t}$, and past experience of the UAV, leading to a sequential decision-making problem under uncertainty. The interplay between $\eta_{t}$ and $p_{t}$ is complex. If $\eta_{t}$ is increased, less data needs to be communicated. However, the processing time on the UAV is increased. If $p_{t}$ is increased, the data rate is improved and therefore the energy consumption is increased. To achieve the optimal trade-off of these factors, the UAV has to adapt continuously to the stochastic nature of both the channel state and the task sizes in real time. This is a problem suited for reinforcement learning as outlined in the subsequent sections.

## 4. Problem Formulation

We have developed a CMDP formulation to model the sequential nature of the resource allocation problem, while accounting for the hard latency constraint imposed by edge computing applications. Our CMDP incorporates an auxiliary cost function to represent the constraint violation of the latency requirement. We also established that the problem’s inherent characteristics—continuous state and action spaces, unknown and potentially nonstationary transition dynamics, coupled decision variables, and real-time operation requirements—necessitate a model-free learning approach. Table 2 gives the specific description of each term used in problem formulation.

The CMDP formulation is formally defined using the tuple (S,A,P,r,c,γ), where S denotes the state space, A represents the action space, P:S×A×S→[0,1] is the transition probability distribution, *r* is the reward function, $c:S\times A\to R_{\geq 0}$ is the constraint cost function, and γ∈[0,1] is the discount factor. A critical difference from unconstrained MDPs is that feasible policies must not only optimize the rewards but also ensure that the expected cumulative cost satisfies the specified constraints. In the context of wireless resource allocation, such constraints reflect the quality-of-service (QoS) requirements of edge computing applications that impose hard boundaries on acceptable latencies and thus cannot be traded off with other objectives [42]. Our CMDP consists of the following elements.

### 4.1. State Space

The five-dimensional state vector given below captures all information relevant to making the current decision. The state vector includes four continuous-valued elements and one categorical element.

(14) $$s_{t}=\left[L_{t}^{\mathrm{norm}},\;\Gamma_{t}^{\mathrm{norm}},\;\eta_{t-1},\;D_{t-1}^{\mathrm{norm}},\;c_{t-1}^{\mathrm{norm}}\right]\in {\left[0,1\right]}^{5}$$

$L_{t}^{\mathrm{norm}}$ is the current task size and normalized by min-max scaling. $\Gamma_{t}^{\mathrm{norm}}$ is the current channel SNR in the normalized form. $\eta_{t-1}$ is the previous computation partitioning ratio. $D_{t-1}^{\mathrm{norm}}$ is the feedback about previous performance obtained by checking the previous latency value. $c_{t-1}^{\mathrm{norm}}$ is the magnitude of the prior constraint violation. The previous latency and cost information is quite useful in the agent knowing about the constraint satisfaction. It actually makes the agent aware of the near- or beyond-feasibility boundary.

### 4.2. Action Space

The action vector controls both local processing and remote processing. The first element of the action vector computation partitioning ratio $\eta_{t}$ determines the portion of the task executed locally, as we have discussed above in Section 3.3. Normalized transmit power $p_{t}^{\mathrm{norm}}$ is the second action in our action space and it controls UAV transmission power. The second element determines the portion of the task executed remotely.

(15) $$a_{t}=\left[\eta_{t},\;p_{t}^{\mathrm{norm}}\right]\in {\left[0,1\right]}^{2}$$

(16) $$p_{t}=P_{\min}+p_{t}^{\mathrm{norm}}\cdot \left(P_{\max}-P_{\min}\right)$$

Both of these continuous actions empower fine-grained control over the energy–latency trade-off. It differentiates our action spaces from discrete action spaces that can miss optimal operating points, especially where the channel condition is variable.

### 4.3. Reward Function

The objective is to decrease and stabilize the amount of energy used to increase the length of time that the UAV will stay aloft and fulfill the requirements of the latency. The immediate reward is defined as the negative of the normalized energy use at any particular moment:

(17) $$r_{t}=-\frac{E_{\mathrm{total},t}}{E_{\mathrm{ref}}}$$

Here, $E_{\mathrm{ref}}$ is a reference energy (a constant number representing the average energy per action taken using a random policy), which helps stabilize the numbers during the training process. Using the components of the energy model as outlined in Section 3.5, the reward is defined as

(18) $$r_{t}=-\frac{1}{E_{\mathrm{ref}}}\left(P_{\mathrm{sense}}T_{\mathrm{sense}}+\zeta f_{\mathrm{UAV}}^{2}\eta_{t}L_{t}\kappa +p_{t}\cdot \frac{\left(1-\eta_{t}\right)L_{t}}{R_{t}}\right)$$

By maximizing cumulative rewards over time, the agent develops an ability to minimize the total amount of energy spent for the entire duration of the mission.

*Constraint Cost Function:*

To meet this latency constraint, we create a cost function to measure when deadlines are missed:

(19) $$c_{t}=\max\left(0,\;D_{t}-T_{\max}\right)$$

We can see that there are at least three advantages of this approach: (1) the cost is either zero or greater than zero as it cannot be negative; (2) costs for all actions that are considered possible are zero; (3) the larger the violation in time from meeting a deadline, the larger the cost of such a violation will be. This provides a continuous increase in the direction of feasibility. This is achieved by expanding the cost function to incorporate the values from the latency model [43].

(20) $$c_{t}=\max\left(0,\;T_{\mathrm{sense}}+\max\left(\frac{\eta_{t}L_{t}\kappa }{f_{\mathrm{UAV}}},\;\frac{\left(1-\eta_{t}\right)L_{t}}{R_{t}}+\frac{\left(1-\eta_{t}\right)L_{t}\kappa }{f_{\mathrm{RSU}}}\right)-T_{\max}\right)$$

### 4.4. Optimization Objective

The optimization problem aims to find a policy π that maximizes the cumulative reward. It is subject to the constraint on the expected cumulative cost.

(21) $$\begin{array}{cc} \underset{\pi }{\mathrm{maximize}}\; & J_{R}\left(\pi \right)\\ \mathrm{subject}\;\mathrm{to}:\; & J_{C}\left(\pi \right)\leq d \end{array}$$

where $J_{R}\left(\pi \right)$ represents the expected cumulative reward, $J_{C}\left(\pi \right)$ represents the expected cumulative cost, and *d* represents the constraint threshold.

(22) $$J_{R}\left(\pi \right)=E_{\pi }\left[\sum_{t=0}^{\infty }\gamma^{t}r_{t}\right]$$

(23) $$J_{C}\left(\pi \right)=E_{\pi }\left[\sum_{t=0}^{\infty }\gamma^{t}c_{t}\right]$$

For d≥0, where *d* is a constraint threshold, when *d* ≈ 0, there will be nearly no tolerance for violating any of the latency constraints because of how critical some types of applications are with regard to their latency. Also, γ=0.99 is used as a discount factor to promote long-term thinking but to ensure that these infinite summations are convergent. The constraint on the cumulative cost in (21) bounds the expected cumulative cost over all tasks, not just individual violations. It is possible for the number of violations of each task to be greater than zero for many tasks (this may simply be a matter of physical feasibility given the channel conditions); however, the long-run average violation rate for each task will be minimized.

### 4.5. Problem Characteristics

This CMDP model exhibits several characteristics that make it difficult to solve using classical methods. The primary issues are: (i) continuous state and action space (hence, table-based methods cannot be used); (ii) uncertainty and variability of the environment (both due to the randomness of the wireless channel and because of the arrival of new tasks); (iii) coupled decisions (decisions regarding splitting of tasks and transmission power affect each other, and are critical in achieving the delay bounds); and (iv) time constraints (decisions must be made rapidly, on the order of milliseconds, thus making slow computational processes unfeasible). Therefore, we require a method that can learn from direct experience and does not require prior knowledge of the environment’s behavior. Thus, we apply a model-free deep reinforcement learning technique. However, since we aim to minimize both delay and energy consumption, we will develop novel model-free policy learning based on Lagrangian dual optimization. The algorithm iteratively performs two main types of steps: initially, the agent learns a policy through trial and error in the environment; secondly, the agent modifies an additional parameter (the Lagrange multiplier λ) such that the delay constraint is satisfied. Through repeated iterations of the two-step process, the agent develops a suitable policy that minimizes energy usage and satisfies the delay constraints required by edge computing applications.

## 5. Research Methodology

This section presents our solution approach for the CMDP formulated in Section 4. We first introduce the Lagrangian relaxation technique that transforms the constrained problem into a tractable form, and then develop the C-SAC algorithm that combines maximum-entropy reinforcement learning with adaptive constraint handling [44]. Two baseline methods have also been described for comparative evaluation.

The proposed approach addresses the key challenges identified in Section 4.5 through a principled combination of stochastic policy optimization and dual constraint handling. By leveraging the maximum-entropy framework, our method maintains exploration throughout training while the Lagrangian formulation ensures systematic constraint satisfaction. This combination is particularly effective for the UAV-MEC environment, where channel stochasticity demands robust policies and latency constraints require explicit enforcement mechanisms.

Table 3 summarizes the hyperparameters used across all algorithms.

### 5.1. Lagrangian Relaxation

Optimizing the objective of the Constrained MDP in (21) is difficult since traditional RL is based on unconstrained reward maximization. The best way to formulate the constrained problem is by using Lagrangian relaxation. This method uses a dual variable that represents a penalty that is used to discourage constraint violations. The Lagrangian function is the combination of the reward objective and a weighted penalty on the constraint cost, and is expressed as follows:

(24) $$L\left(\pi ,\lambda \right)=J_{R}\left(\pi \right)-\lambda \left(J_{C}\left(\pi \right)-d\right)$$

where λ≥0 denotes the Lagrange multiplier (dual variable). Expanding the above objective yields

(25) $$L\left(\pi ,\lambda \right)=E_{\pi }\left[\sum_{t=0}^{\infty }\gamma^{t}\left(r_{t}-\lambda c_{t}\right)\right]+\lambda d$$

The original constrained problem is equivalent to finding the saddle point:

(26) $$\max_{\pi }\min_{\lambda \geq 0}\;L\left(\pi ,\lambda \right)$$

The min-max formulation has an intuitive interpretation: the policy π wants to maximize the rewards it receives while minimizing the constraint costs; meanwhile, λ is acting like an adaptive penalty that gets larger if the constraints are broken and smaller if they are met. We can interpret the Lagrange multiplier λ as being the price of violating a constraint. If λ is large, the policy is going to focus on reducing latency; if λ is small, then the policy will be more concerned with energy efficiency. Rather than having to manually tune this trade-off, our algorithm will learn λ automatically through the process of optimizing the dual objective through gradients.

### 5.2. Maximum-Entropy Framework

The primary goal of standard reinforcement learning is to identify policies which achieve the highest expected accumulated reward. Maximum-entropy reinforcement learning (RL) introduces an additional “entropy bonus” to the primary goal of maximizing the expected accumulated reward to encourage exploration and increase robustness.

(27) $$J_{\mathrm{MaxEnt}}\left(\pi \right)=E_{\pi }\left[\sum_{t=0}^{\infty }\gamma^{t}\left(r_{t}+\alpha H\left(\pi \left(\cdot |s_{t}\right)\right)\right)\right]$$

where $H\left(\pi \left(\cdot |s\right)\right)=-E_{a∼\pi }\left[\log\pi \left(a|s\right)\right]$ is the policy entropy and α>0 is the temperature parameter that controls the trade-off between exploration and exploitation. Two key benefits exist from this approach to our particular problem. First, the probabilistic nature of the policy provides a means of handling the uncertainty present in wireless communications—rather than selecting one specific action, the policy selects a distribution that can provide protection against potential variations in the communication channel. Second, by introducing an entropy penalty, we are able to prevent the agent’s policy from prematurely converging to a less optimal deterministic strategy, thereby allowing the agent to discover more optimal, and therefore more robust, solutions.

### 5.3. Constrained Soft Actor–Critic (C-SAC)

The Constrained Soft Actor–Critic algorithm is proposed in this paper, and it extends the maximum-entropy framework so that it is able to treat explicit constraints as part of its decision-making process using a Lagrangian dual optimization technique. The Constrained SAC incorporates three main features: (i) entropy-regularized policy optimization for robust exploration, (ii) a constraint critic to anticipate long-term constraint violations, and (iii) an adaptive Lagrange multiplier to allow for automatic tuning of constraints.

#### 5.3.1. Network Architecture

C-SAC utilizes trained neural networks—an actor, two reward critics, and a constraint critic. All networks are implemented as a multi-layer perceptron with [256, 256] hidden layers and ReLU activations, with each critic additionally having a target network updated via soft updates for training stability. Actor network $\pi_{\phi }\left(a|s\right)$ outputs a Gaussian distribution over actions. Given state s, it produces mean $\mu_{\phi }\left(s\right)$ and log-standard-deviation $\log\sigma_{\phi }\left(s\right)$, from which actions are sampled using the reparameterization trick. Twin reward critics $Q_{\theta_{1}}\left(s,a\right)$, $Q_{\theta_{2}}\left(s,a\right)$ estimate the expected cumulative reward. Using two critics and taking their minimum mitigates overestimation bias common in actor–critic methods [45]. Constraint critic $Q_{\psi }^{C}\left(s,a\right)$ estimates the expected cumulative constraint cost:

(28) $$Q_{\psi }^{C}\left(s,a\right)\approx E_{\pi }\left[\sum_{k=0}^{\infty }\gamma^{k}c_{t+k}|s_{t}=s,a_{t}=a\right]$$

**Figure 2:**
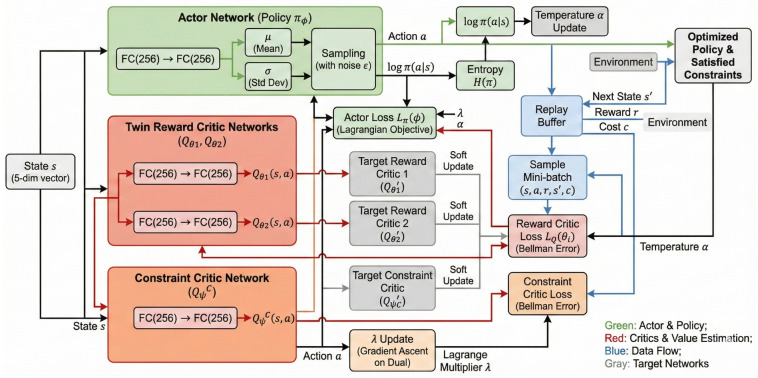
Neural network architecture of the C-SAC algorithm. The actor network outputs a Gaussian policy over continuous actions. Twin reward critics estimate the expected discounted return to mitigate overestimation bias, while the constraint critic estimates the expected discounted constraint cost used for Lagrangian-based constraint enforcement.

This dedicated network enables the agent to anticipate long-term constraint violations rather than reacting only to immediate costs. Figure 2 shows the working principle of the C-SAC algorithm. Each critic has a corresponding target network (updated via soft updates) that provides stable learning targets.

#### 5.3.2. Constrained Policy Objective

Combining the maximum-entropy objective with Lagrangian constraint handling, C-SAC optimizes:

(29) $$L_{C-\mathrm{SAC}}\left(\pi ,\lambda \right)=E_{\pi }\left[\sum_{t=0}^{\infty }\gamma^{t}\left(r_{t}+\alpha H\left(\pi \left(\cdot |s_{t}\right)\right)-\lambda c_{t}\right)\right]+\lambda d$$

This integrates three components: reward maximization for energy efficiency, entropy regularization for robust exploration, and constraint cost penalization for latency compliance. The balance among these objectives is determined by α and λ, both learned automatically during training.

#### 5.3.3. Learning Updates

At each training step, the algorithm samples a mini-batch from the replay buffer and performs the updates outlined below. These updates are performed sequentially, with each component contributing to the overall learning objective. The mini-batch sampling from the replay buffer enables off-policy learning, allowing the algorithm to reuse past experiences for improved sample efficiency.

**Reward Critic Update.** The critics are trained by minimizing the Bellman residual:

(30) $$L_{Q}\left(\theta_{i}\right)=E_{\left(s,a,r,s^{\prime }\right)}\left[{\left(Q_{\theta_{i}}\left(s,a\right)-y^{R}\right)}^{2}\right],\;i\in \left\{1,2\right\}$$

The use of twin critics, where the minimum Q-value is taken, helps mitigate the overestimation bias that commonly affects actor–critic algorithms, where the target value incorporates the entropy bonus:

(31) $$y^{R}=r+\gamma \left(\min_{i=1,2}Q_{{\bar{\theta }}_{i}}\left(s^{\prime },a^{\prime }\right)-\alpha \log\pi_{\phi }\left(a^{\prime }|s^{\prime }\right)\right),\;a^{\prime }∼\pi_{\phi }\left(\cdot |s^{\prime }\right)$$

**Constraint Critic Update.** The constraint critic minimizes a similar loss:

(32) $$L_{Q^{C}}\left(\psi \right)=E_{\left(s,a,c,s^{\prime }\right)}\left[{\left(Q_{\psi }^{C}\left(s,a\right)-y^{C}\right)}^{2}\right]$$

with target

(33) $$y^{C}=c+\gamma \;Q_{\bar{\psi }}^{C}\left(s^{\prime },a^{\prime }\right),\;a^{\prime }∼\pi_{\phi }\left(\cdot |s^{\prime }\right)$$

It is also important to note that the constraint target does not include the entropy bonus since the entropy regularizer can be applied only to the reward function. This separation ensures that the constraint critic provides an unbiased estimate of future constraint violations, which is essential for accurate Lagrangian penalty computation.

**Actor Update.** The policy is updated to maximize the Lagrangian objective:

(34) $$L_{\pi }\left(\phi \right)=E_{s,a∼\pi_{\phi }}\left[\alpha \log\pi_{\phi }\left(a|s\right)-Q_{\theta_{1}}\left(s,a\right)+\lambda Q_{\psi }^{C}\left(s,a\right)\right]$$

The loss is meant to encourage actions that achieve high rewards, low constraint costs, and enough entropy to allow for exploration.

**Temperature Update.** The entropy coefficient α is adjusted to maintain a target entropy level $\bar{H}=-\dim\left(A\right)$:

(35) $$\alpha \leftarrow \alpha -\alpha_{\alpha }\nabla_{\alpha }E_{s,a∼\pi_{\phi }}\left[-\alpha \left(\log\pi_{\phi }\left(a|s\right)+\bar{H}\right)\right]$$

**Lagrange Multiplier Update.** The dual variable λ is updated via gradient ascent:

(36) $$\lambda \leftarrow \mathrm{clip}\left(\lambda +\alpha_{\lambda }\left(E_{s,a∼\pi_{\phi }}\left[Q_{\psi }^{C}\left(s,a\right)\right]-d\right),\;0,\;\lambda_{\max}\right)$$

λ will increase when the estimated constraint cost exceeds a given threshold of *d*. This in turn will amplify the penalty for taking an action that violates the constraint. Conversely, when λ is decreased because the estimated constraint cost is below the threshold of *d*, this means the cost of the last action taken was within the constraint. In either case, the clipping helps ensure that λ is always non-negative and has a finite value so as to promote numerical stability.

**Target Network Update.** All target networks are updated via an exponential moving average with coefficient τ:

(37) $$\bar{\theta }\leftarrow \tau \theta +\left(1-\tau \right)\bar{\theta }$$

This soft update mechanism provides stable learning targets, preventing the oscillations that can occur when target networks are updated abruptly. The small value of τ ensures gradual target network changes while still tracking the learning progress of the main networks.

The reinforcement learning hyperparameters follow established practices for stable, sample-efficient learning. We use a near-unity discount factor (γ=0.99) for long-horizon planning and conservative learning rates ($\alpha_{\pi },\alpha_{Q}=3\times 10^{-4}$) for stable updates, as per SAC conventions [44]. The Lagrange multiplier learning rate ($\alpha_{\lambda }=3\times 10^{-3}$) is set higher for responsive constraint handling, a choice validated by our ablation study (Section 6.6) [46]. All neural networks (actor, reward critics, and constraint critic) employ a multi-layer perceptron (MLP) architecture consisting of two hidden layers, each containing 256 neurons (denoted as 2×256 in Table 3). The input layer receives the 5-dimensional state vector, while the output layer produces either action distribution parameters (for the actor, outputting mean and log-standard deviation for each of the 2 action dimensions) or scalar value estimates (for the critics). ReLU activation functions are applied after each hidden layer to introduce non-linearity. This architecture provides sufficient capacity without excessive computation [45], and the target entropy is automatically set to −dim(A)=−2, a standard heuristic for this action space [44].

The complete algorithm is presented in Algorithm 1.
**Algorithm 1:** Constrained Soft Actor–Critic (C-SAC)
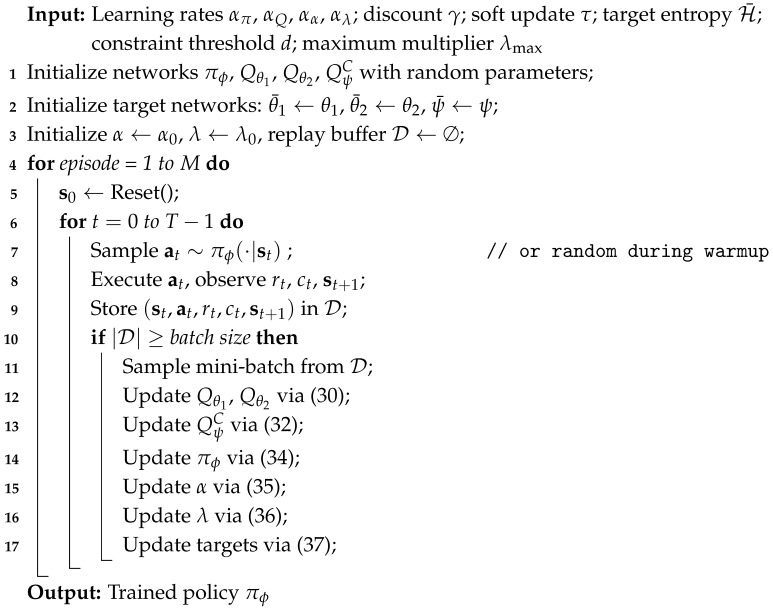


### 5.4. Baseline Algorithms

To evaluate the effectiveness of our approach, we compare it against two baselines representing alternative design choices.

**Soft Actor–Critic (SAC).** The unconstrained variant of our algorithm, which optimizes only the maximum-entropy objective without explicit constraint handling [44]. SAC uses identical network architecture and hyperparameters but lacks the constraint critic $Q_{\psi }^{C}$ and Lagrange multiplier λ. Any constraint awareness must emerge implicitly through reward shaping, which we do not employ. This baseline tests whether explicit constraint handling is necessary.

**TD3-Lagrangian.** A constrained variant of Twin Delayed DDPG that uses deterministic policies with Lagrangian constraint handling [45]. Unlike the stochastic policies in SAC and C-SAC, TD3 outputs deterministic actions with added exploration noise during training. This baseline tests whether stochastic policies offer advantages in our high-variance channel environment. The implementation follows the original TD3 with the addition of a constraint critic and Lagrange multiplier update identical to C-SAC.

Their main differences are summarized as follows: C-SAC and SAC both employ stochastic Gaussian policies with entropy regularization, while TD3-Lagrangian uses deterministic policies with additive noise for exploration. On the other hand, C-SAC and TD3-Lagrangian both incorporate Lagrangian constraint handling, whereas SAC relies only on reward maximization. These design choices enable us to distinguish the contributions of (i) explicit constraint handling and (ii) stochastic versus deterministic policy architectures.

## 6. Simulation Results

In this section, we will evaluate our proposed C-SAC method against the following baselines: (1) the unconstrained SAC (Soft Actor–Critic); (2) deterministic TD3-Lagrangian. Firstly, we explain the experimental setting and provide an analysis of training dynamics, policy behavior, and overall performance. In addition, ablation studies and sensitivity studies are provided to test our design decisions under different settings.

### 6.1. Experimental Setup

All experiments were conducted for 500 training epochs with each epoch having 200 time steps. The simulation parameters in Table 3 reflect the UAV-assisted edge computing environment as characterized in the literature. The simulation environment was implemented in Python (3.11.9) using PyTorch (2.9.1) for neural network training. To reflect real intelligent transportation workloads, the mean task size ($\mu_{L}=5$ Mbits) and computational intensity (κ=1500 cycles/bit) are set based on typical edge tasks like object detection [36]. The 10:1 ratio between edge server ($f_{\mathrm{RSU}}=20$ GHz) and UAV processor ($f_{\mathrm{UAV}}=2$ GHz) frequencies justifies offloading and aligns with commercial hardware capabilities [18]. The stringent latency deadline ($T_{\max}=35$ ms) is derived from URLLC and autonomous vehicle requirements [22].

The channel is configured with standard 5G bandwidth (B=20 MHz) [37]. A mean SNR of $\bar{\Gamma }=10$ dB with $\sigma_{\Gamma }=5$ dB deviation represents realistic, variable air-to-ground links [21,26]. The transmit power range ($P_{\min}=0.01$ W, $P_{\max}=1$ W) models practical UAV power constraints [18].

### 6.2. Training Convergence and Constraint Satisfaction

**Figure 3:**
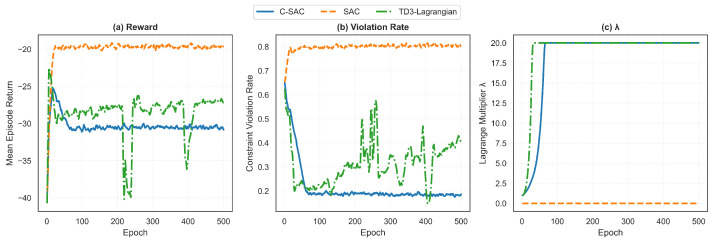
Learning curves: (**a**) mean episode return as a measure of energy usage; (**b**) constraint violation rate; (**c**) Lagrangian multiplier evolution. The Constrained SAC achieved the most stable convergence to the smallest violation rate of all methods tested, whereas TD3-Lagrangian had continued instability.

The training curves are shown in Figure 3, which demonstrates fundamental differences between these methods in their trade-offs between energy usage and constraint violation rates.

#### 6.2.1. Reward and Violation Trade-Off

SAC has the highest return (approximately −20), which it obtains by using no constraint knowledge and very aggressive reduction in energy use; however, SAC’s large energy savings are offset by violations of 79%. This is why SAC is considered unsuitable for latency-critical applications. C-SAC achieves a return of roughly −31, representing significantly more energy usage than SAC and constraint violations of 18.9%. TD3-Lagrangian’s performance is somewhere in between C-SAC and SAC, with constraint violations of about 41%; however, there are some oscillations in TD3-Lagrangian’s training path. There is clear stability in the return and latency violation ratio of C-SAC. The most important finding is that the extra energy that C-SAC uses (about 57% more than SAC) represents the cost of having satisfied the constraint reliably. If the application has consequences as severe as those resulting from violations of deadlines, then this trade-off is considered favorable [38]. C-SAC has a significant advantage over the baselines:

(38) $$\begin{array}{cc} \mathrm{Violation}\;\mathrm{Reduction}\;\mathrm{vs}.\;\mathrm{SAC} & =\frac{79.5\%-18.9\%}{79.5\%}=76.2\%\\ \mathrm{Violation}\;\mathrm{Reduction}\;\mathrm{vs}.\;\mathrm{TD}3-\mathrm{Lag} & =\frac{41.3\%-18.9\%}{41.3\%}=54.2\% \end{array}$$

#### 6.2.2. Lagrange Multiplier Dynamics

As we can see in Figure 3c, both constrained methods increase λ rapidly in the initial training phases and they reach a maximum λ value of $\lambda_{\max}=20$ by epoch 100. The maximum λ value shows the high activity of the constraint; the algorithms will always use the maximum penalty pressure. There is a big difference in how the algorithm responds to this penalty. C-SAC transforms this penalty into constant constraint satisfaction. In contrast, TD3-Lagrangian’s deterministic policy does not adequately respond to this penalty and therefore continues to have constraint violations, even though the same penalty magnitude is applied. This fundamental difference in stability stems from the mismatch between TD3-Lagrangian’s deterministic policy and the highly stochastic UAV-MEC environment. A deterministic policy outputs a single fixed action for each state, making it inherently brittle when the wireless channel exhibits significant randomness. During training, TD3-Lagrangian occasionally encounters channel conditions where its deterministic action leads to severe constraint violations. However, because the policy provides no distributional coverage over alternative actions, it cannot hedge against uncertainty. When the policy updates in response to these violations, it can overcorrect—shifting dramatically to avoid the problematic state–action pairs. This creates oscillations where the policy alternates between overly conservative (high η, minimal offloading) and overly aggressive (low η, maximum offloading) strategies. C-SAC’s stochastic policy mitigates this instability through two mechanisms: (1) the policy outputs a distribution over actions, naturally hedging against channel uncertainty by maintaining diverse action candidates, and (2) the entropy regularization explicitly discourages premature commitment to deterministic strategies, preserving exploration throughout training. This distributional coverage allows C-SAC to smoothly adapt to constraint violations without the abrupt policy shifts that characterize deterministic methods.

**Figure 4:**
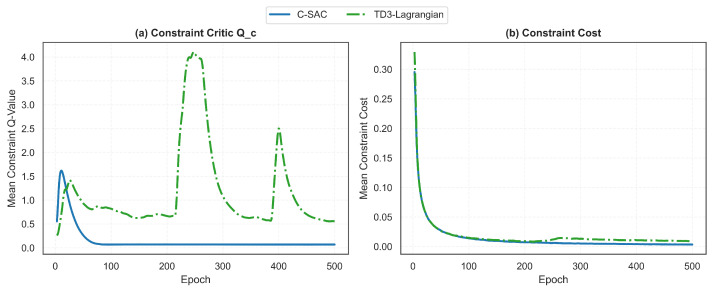
Constraint critic analysis: (**a**) mean predicted constraint Q-value $Q^{C}$, and (**b**) actual constraint cost during training. C-SAC’s constraint critic converges smoothly, while TD3-Lagrangian exhibits large oscillations.

The constraint critics shown in Figure 4 give additional insight into learning constraints from data.

The C-SAC constraint critic $Q^{C}$ is constrained to a limit below 0.05 by epoch 100 from its starting point of 1.6 and then remains relatively steady. The smoothness of this convergence indicates that it has learned the constraint cost function well. In stark contrast, the constraint critic for TD3-Lagrangian shows large spikes up to 4.0 at epochs 250 and 400, which aligns with the periodic violations shown in Figure 3b. These fluctuations are due to difficulties in estimating constraint costs based on the deterministic nature of the policy over the stochastic state space—something that stochastic policies can inherently overcome through their distributional coverage.

### 6.3. Channel-Adaptive Policy Behavior

**Figure 5:**
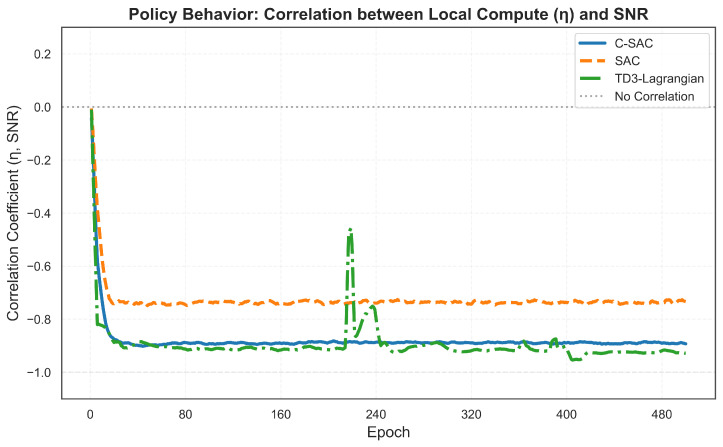
Evolution of correlation coefficient between η and SNR during training. Negative correlation indicates channel-adaptive behavior: lower η (more offloading) when channel conditions are favorable.

A central finding of this work is that the learned policies exhibit strong channel-adaptive behavior without explicit channel modeling in the reward function. Figure 5 shows the Pearson correlation coefficient between the local computation ratio η and instantaneous SNR over training.

The optimal strategy is a negative correlation: when SNR is high, the agent should reduce η to offload more computation, exploiting the favorable channel for efficient transmission. Conversely, poor channel conditions favor local processing to avoid transmission delays. All three algorithms discover this strategy, but with varying degrees of consistency:

- **C-SAC** (ρ=−0.894): Achieves strong, stable correlation by epoch 80 with minimal variance thereafter.
- **TD3-Lagrangian** (ρ=−0.928): Achieves the strongest final correlation but exhibits instability, with spikes toward −0.4 at epochs 220 and 400.
- **SAC** (ρ=−0.736): Shows weaker adaptation, as the unconstrained objective provides less incentive for latency-aware decisions.

**Figure 6:**
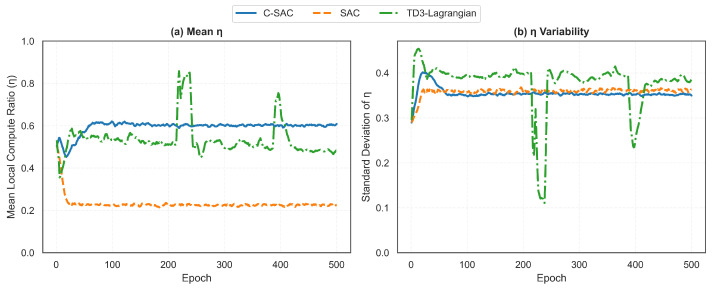
Statistics of the local computation ratio: (**a**) mean η value, and (**b**) standard deviation reflecting action variability.

A deceptive inconsistency emerges: TD3-Lagrangian attains stronger η-SNR correlation yet has low constraint satisfaction. This arises because correlation measures average behavior, while constraint satisfaction depends on worst-case performance. TD3-Lagrangian’s deterministic policy sometimes overreacts to channel variations, causing the spikes visible in Figure 5. During these instability periods, the policy temporarily loses its adaptive behavior, leading to violation bursts. The stochastic policy of C-SAC provides more consistent adaptation but, slightly weaker in correlation scale, it results in better overall constraint satisfaction [47]. Figure 6 further characterizes the action distributions.

C-SAC has $\bar{\eta }\approx 0.61$, which means that it is in favor of local computing as much as possible to have some buffer for channel uncertainty. The SAC has a very aggressive offloading strategy ($\bar{\eta }\approx 0.22$) and accepts high-latency risk so that it can minimize its local computing power consumption. TD3-Lagrangian is somewhere in the middle ($\bar{\eta }\approx 0.48$), but there are significant spikes up to 0.85 during unstable time periods. The standard deviation plot shows another important aspect of how the algorithms behave differently. C-SAC and SAC have a consistent level of variability ranging from 0.35 to 0.40; this suggests that they have a good and consistent balance between exploration and exploitation. The standard deviation of TD3-Lagrangian drops to 0.10 during times of instability (epochs 220–280, 380–420); this indicates that the algorithm temporarily collapsed into nearly deterministic behavior. This collapse into nearly deterministic behavior would explain why the violation spikes occur: when the action space is limited by reduced action variety, the algorithm cannot effectively respond to changes in the channels.

### 6.4. Comparative Performance Analysis

**Figure 7:**
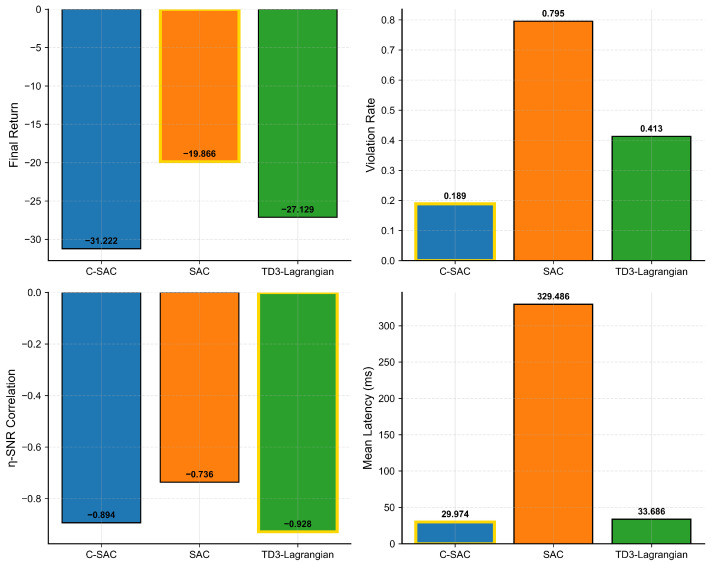
The gold border is used to highlight which algorithm performed the best on each of the metrics in this figure. The C-SAC outperformed all other constraints for both constraint violation and latency, but SAC achieved better energy performance than the other 2 algorithms at the expense of violating many of the constraints.

Figure 7 represents a summary of the final performance results from all of the algorithms. Table 4 provides the numerical values.

Here are some points for discussion:

**Constraint Satisfaction.** C-SAC experiences 18.9% violations, which means that it satisfies over 81% of its tasks within the 35 ms deadline. Compared to SAC and TD3-Lagrangian, C-SAC has achieved a 4.2× better performance as compared to SAC in terms of constraint satisfaction and a 2.2× better performance compared to TD3-Lagrangian. The comparison highlights the significant advantage of using explicit constraint handling via Lagrangian methods over implicit constraint handling methods.

**Latency Performance.** C-SAC produces a mean latency of 29.97 ms, which is 14% under the 35 ms deadline. In contrast, TD3-Lagrangian produces a mean latency of 33.69 ms with a 4% margin to the deadline. Finally, SAC has an extremely high mean latency of 329 ms (approximately 10× the deadline), demonstrating how completely SAC ignores time constraints.

**Trade-Off Between Energy and Constraints.** C-SAC uses about 57% more energy than SAC. As such, whether or not the additional energy use is acceptable will depend on the specific needs of each application scenario. For instance, if there are mission-critical applications that cannot afford to violate deadlines, then the increased energy use by C-SAC can be justified. The increase in energy usage is primarily due to C-SAC being more conservative. It uses higher average η (which means it uses more local processing), and it transmits more power when it needs to offload reliably.

### 6.5. Energy–Constraint Trade-Off Analysis

**Figure 8:**
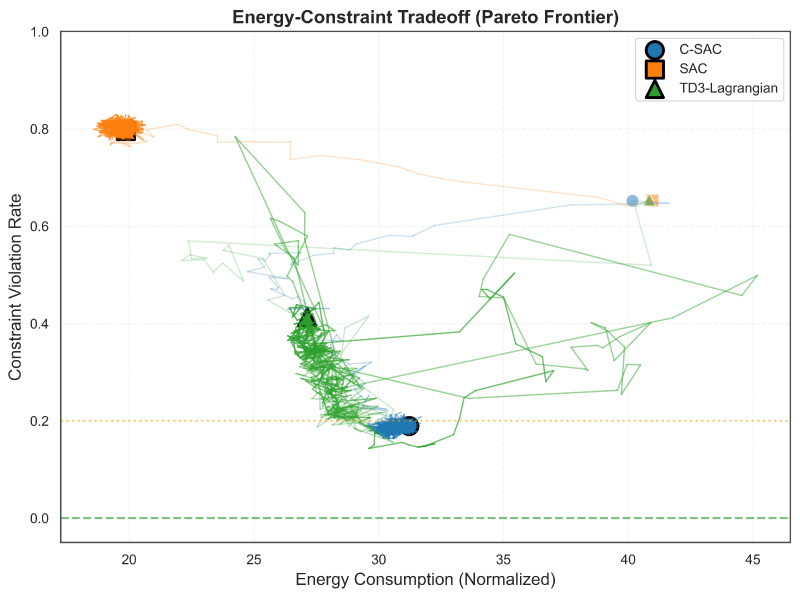
Training trajectories in energy–violation space. Large markers indicate final converged positions. The dashed lines show zero violations (ideal) and 20% threshold.

Figure 8 visualizes the training trajectories in the energy–violation space, revealing how each algorithm navigates the fundamental trade-off.

SAC has been unable to escape from the high-violation, low-energy corner for most of the training process, due to the fact that SAC’s objective does not provide an incentive to decrease violation levels. Both of the constrained methods moved to lower violation levels, but they have very different properties. C-SAC moved along a generally straight-line path to a small cluster centered at (31, 19%), while the path taken by TD3-Lagrangian was more irregular, and explored a much larger area of space, eventually coming to rest near (27, 41%) with large variability in both dimensions. The better trade-off offered by C-SAC’s final position compared to TD3-Lagrangian’s final position is particularly noteworthy: for about 15% more energy, C-SAC achieved over 50% more violations reduced. This may indicate that the stochastic policy used by C-SAC has found a more efficient operating point on the constraint boundary defined by the energy constraint. In addition, the more tightly clustered final positions of C-SAC demonstrate more stable behavior upon convergence.

### 6.6. Ablation Study

We performed the ablation study using the same testbed as for the evaluation of C-SAC’s performance in order to assess how individual components contribute to the overall performance. We disabled or modified important parts of the mechanism, as illustrated in Table 5.

#### 6.6.1. Adaptive Lagrange Multiplier

A reduction in the learning rate $\alpha_{\lambda }$ of 10×, which affects how quickly the constraints can be updated, increases the number of constraint violations from 18.2% to 44.4%. It also allows the policy to reach a constraint-violating equilibrium because the slow update speed does not allow λ to be sufficiently large in the early stages of training. An increase in $\alpha_{\lambda }$ of 10× produces little change, showing that the chosen value of the learning rate was sufficient. Thus, the adaptive nature of the mechanism has an asymmetric effect on the learning process, and should therefore be tuned to have a faster adaptation of the penalty. This phenomenon occurs because the Lagrange multiplier λ acts as a dynamic penalty weight on constraint violations. When $\alpha_{\lambda }$ is too small, λ increases too slowly during early training when the policy is still learning. Consequently, the constraint cost penalty remains weak relative to the energy reward, allowing the policy to settle into a local optimum that prioritizes energy minimization over latency satisfaction. Once stabilized at this equilibrium, the small learning rate prevents λ from increasing rapidly enough to escape the constraint-violating region. In practical terms, this is analogous to a control system with insufficient gain—the feedback signal (constraint violations) is detected but the corrective action (increasing λ) is too weak to drive the system toward the desired operating point (constraint satisfaction). The adaptive mechanism with appropriately tuned $\alpha_{\lambda }=3\times 10^{-3}$ avoids this by enabling rapid penalty escalation during initial training, preventing the policy from converging prematurely to infeasible solutions. Even a manually selected value of λ=15, which could produce good results, produced only 20.4% constraint violation compared to 18.2% when using the adaptive mechanism. Therefore, the adaptive mechanism provides a significant practical advantage over using fixed values of λ [46], as there is no need for manual searches for suitable values of λ.

#### 6.6.2. Adaptive Entropy Temperature

In removing the ability of the entropy temperature to automatically adjust, its parameters degrade both the constraint violation and channel adaptation. Using a fixed value of α=0.1 (too small), the policy converged too quickly and achieved 24.1% constraint violations with a significantly reduced η-SNR correlation (−0.761 vs. −0.887). Using a fixed value of α=0.5 (too big) resulted in too much randomness in the policy and prevented reliable convergence, achieving 39.5% constraint violations with a severely degraded η-SNR correlation (−0.601). As demonstrated in Figure 3, the adaptive mechanism of the entropy temperature converged to α≈0.025. This represents the optimal point at which to balance exploration and exploitation. This automatic tuning eliminates a sensitive hyperparameter that would otherwise need problem-specific modification.

### 6.7. Sensitivity Analysis

In this section, we examine how well our algorithms can perform in different environments. A three-dimensional analysis is performed: deadline tolerance, the sensitivity of the algorithms to changes in channel characteristics, and sensitivity to the value of Lagrange multipliers.

#### 6.7.1. Deadline Sensitivity

The results of deadline sensitivity change are shown in Table 6. The table presents constraint violation percentages as deadlines vary from a tight deadline of 25 ms to a relaxed deadline of 55 ms.

C-SAC demonstrates gradual degradation of performance as tighter constraints are imposed on the deadline. Although C-SAC experiences a high number of constraint violations (52.4%) when the deadline is set to 25 ms, this is still better than the baselines tested. As deadlines are relaxed, the number of constraint violations experienced by C-SAC decreases monotonically to 4.6% at 55 ms. This predictable relationship between the deadline and performance makes C-SAC suitable for applications with varying latency requirements. Regardless of the deadline, SAC continues to experience constraint violations at rates greater than 65%, confirming that an unconstrained version of SAC is fundamentally unsuited for use in latency-sensitive applications. TD3-Lagrangian appears to scale reasonably well; however, at 55 ms, TD3-Lagrangian displays an anomaly where it achieves a low constraint violation rate of 16.7%, but at the same time, its η-SNR correlation collapses to −0.400. This indicates that the policy learned by TD3-Lagrangian has adopted a near-constant strategy rather than one which adapts to the changing environment. Thus, we have demonstrated another common failure mode of deterministic strategies under easy-to-satisfy constraints [48].

#### 6.7.2. Channel Variability Sensitivity

The results of channel sensitivity are presented in Table 7. In these tests, we examined how our algorithms respond to different levels of channel fading by varying $\sigma_{\Gamma }$ over the values of 4 dB (a mild level of fading), 8 dB (an average level of fading), and 12 dB (a severe level of fading).

C-SAC demonstrates remarkable robustness: as channel variability triples, the violation rate increases by only 1.9 percentage points (17.1% to 19.0%). This stability arises from the stochastic policy’s inherent hedging against uncertainty—by maintaining action diversity, C-SAC performs well across the distribution of possible channel realizations [47].

TD3-Lagrangian exhibits non-monotonic sensitivity with a 20.1-percentage-point range. Interestingly, it performs best under moderate fading (27.8% at $\sigma_{\Gamma }=8$ dB) and worst under severe fading (47.9% at 12 dB). Moderate variability may provide beneficial curriculum learning during training, while severe variability overwhelms the deterministic policy’s limited exploration capacity.

#### 6.7.3. Lagrange Multiplier Bound

The study in Table 8 demonstrates how changes in $\lambda_{\max}$ affect the balance between energy and violation constraints.

An increase in $\lambda_{\max}$ allows for greater constraint enforcement but comes at the price of higher energy consumption. The curve shows that one increasing their lambda values (doubling them) from 50 to 100 will result in a 1.8 percent reduction in violations while increasing their energy usage by 7.3 percent. We conclude based on our study that an optimal value for lambda is $\lambda_{\max}\in \left[20,50\right]$.

### 6.8. Summary of Findings

The experimental evaluation supports the following conclusions:

1. **Explicit constraint handling is essential.** C-SAC reduces violations by 76% compared to unconstrained SAC, demonstrating that implicit reward shaping cannot achieve reliable constraint satisfaction.
2. **Stochastic policies outperform deterministic alternatives.** C-SAC achieves 54% lower violations than TD3-Lagrangian despite using identical Lagrangian constraint handling, with the gap widening under severe channel conditions.
3. **Adaptive mechanisms are critical.** Both adaptive λ and adaptive α contribute significantly; removing either degrades performance by 30–140%.
4. **Channel-adaptive behavior emerges naturally.** All algorithms discover the optimal negative η-SNR correlation without explicit channel modeling, but C-SAC maintains this behavior most consistently.
5. **C-SAC exhibits robust generalization.** Violation rates vary by less than 2 percentage points as channel variability triples, enabling reliable deployment across diverse operating conditions.

## 7. Conclusions

This paper addressed the joint computation partitioning and power allocation problem for UAV-assisted edge computing systems operating under stringent latency constraints. We formulated the resource allocation problem as a Constrained Markov Decision Process and proposed the Constrained Soft Actor–Critic (C-SAC) algorithm, which combines maximum-entropy reinforcement learning with Lagrangian dual optimization for principled constraint handling. The algorithm employs a dedicated constraint critic network to anticipate long-term violations and an adaptive Lagrange multiplier that automatically balances energy efficiency against constraint satisfaction without manual hyperparameter tuning.

Comprehensive experiments demonstrated that C-SAC achieves 76% lower constraint violations compared to unconstrained SAC and 54% improvement over deterministic TD3-Lagrangian, while maintaining a mean latency of 29.97 ms against the 35 ms deadline. The learned policies exhibit strong channel-adaptive behavior with a correlation coefficient of −0.894 between the local computation ratio and channel SNR, despite no explicit channel modeling in the reward function. This emergent behavior demonstrates that deep reinforcement learning can discover sophisticated resource allocation strategies directly from environment interaction. Notably, while TD3-Lagrangian achieved stronger instantaneous channel adaptation, its deterministic nature led to instability under channel uncertainty, confirming that stochastic policies provide inherent robustness valuable for wireless systems with rapidly fluctuating conditions. Ablation studies verified that both the adaptive Lagrange multiplier and automatic entropy tuning are essential, with their removal degrading performance by 30–140%. Sensitivity analyses further showed that C-SAC maintains robust performance across varying channel conditions, with violation rates changing by less than 2 percentage points as channel variability triples.

The current work has several limitations that suggest directions for future research. The formulation assumes a single UAV with fixed hovering position; extending it to multi-UAV scenarios with trajectory optimization would enable coordinated resource allocation and improved coverage. The channel model, while capturing essential fading characteristics, does not account for interference from competing transmissions. Future work could incorporate multi-agent reinforcement learning for cooperative UAV networks, joint trajectory and resource optimization, and transfer learning approaches to reduce training time when deploying to new environments. Hardware testbed validation would provide insights into real-world deployment challenges and computational requirements for onboard policy execution. As sixth-generation wireless networks evolve toward tighter integration of sensing, communication, and computation, the C-SAC framework offers a principled approach to intelligent resource allocation under strict quality-of-service constraints.

## Figures and Tables

**Table 1:** Comparison of existing approaches and our proposed C-SAC.

Reference	Platform	Offloading	Algorithm	Constraint Handling	Stochasticity
Li et al. [18]	UAV	Partial	SCA/Dinkelbach	Implicit (Penalty)	No
Ning et al. [25]	Vehicular	Partial	DRL	Reward Shaping	No
Sun et al. [27]	UAV	Partial	Alt. Opt./KKT/SCA	Implicit (Penalty)	No
Qin et al. [28]	UAV	Partial	Lagrangian/BCD	Implicit (Penalty)	No
Yong et al. [29]	MEC (D2D)	Partial	Alt. Opt. (EPSO-GA)	Implicit (Penalty)	No
Kong et al. [20]	Maritime	Binary	TD3	Reward Shaping	No
Wang et al. [19]	MEC	Binary	TD3	Reward Shaping	No
Liu et al. [33]	Slicing	N/A	CRL	Lagrangian Dual	No
Our Work	UAV	Continuous	C-SAC	Lagrangian Dual	Yes

**Table 2:** Definition of symbols.

Symbol	Definition	Symbol	Definition
*h*	UAV altitude above ground	$E_{\mathrm{sense}}$	Sensing energy consumption
$T_{\max}$	Maximum latency deadline	$E_{\mathrm{comp},t}$	Local computation energy
$L_{t}$	Task data size at time *t*	$E_{\mathrm{comm},t}$	Communication energy
$\mu_{L}$	Mean task size	$E_{\mathrm{total},t}$	Total controllable energy
$\sigma_{L}^{2}$	Variance of task size	$E_{\mathrm{ref}}$	Reference energy for normalization
$L_{\min},L_{\max}$	Task size bounds	S	State space
κ	Computational intensity	A	Action space
*B*	Channel bandwidth	P	Transition probability function
$\Gamma_{t}$	Instantaneous SNR at time *t*	*r*	Reward function
$\bar{\Gamma }$	Mean SNR	*c*	Constraint cost function
$\sigma_{\Gamma }^{2}$	Variance of SNR	γ	Discount factor
$\Gamma_{\min},\Gamma_{\max}$	SNR bounds	$s_{t}$	State at time slot *t*
$p_{t}$	Transmit power at time *t*	$L_{t}^{\mathrm{norm}}$	Normalized task size
$P_{\min},P_{\max}$	Transmit power bounds	$\Gamma_{t}^{\mathrm{norm}}$	Normalized channel SNR
$R_{t}$	Achievable data rate	$D_{t-1}^{\mathrm{norm}}$	Normalized previous latency
$\eta_{t}$	Local computation ratio	$c_{t-1}^{\mathrm{norm}}$	Normalized previous cost
$f_{\mathrm{UAV}}$	UAV processor frequency	$a_{t}$	Action at time slot *t*
$f_{\mathrm{RSU}}$	Edge server frequency	$p_{t}^{\mathrm{norm}}$	Normalized transmit power
$T_{\mathrm{sense}}$	Fixed sensing duration	$r_{t}$	Immediate reward at time *t*
$T_{\mathrm{local},t}$	Local computation time	$c_{t}$	Constraint cost at time *t*
$T_{\mathrm{tx},t}$	Transmission time	π	Policy π:S→P(A)
$T_{\mathrm{edge},t}$	Edge computation time	$J_{R}\left(\pi \right)$	Expected cumulative reward
$D_{t}$	End-to-end latency	$J_{C}\left(\pi \right)$	Expected cumulative cost
$P_{\mathrm{sense}}$	Sensing subsystem power	*d*	Constraint threshold
ζ	Switched-capacitance coeff.		

**Table 3:** Algorithm and simulation parameters.

Parameter	Symbol	Value	Parameter	Symbol	Value
*Algorithm Hyperparameters*			*TD3-Lagrangian-Specific*		
Discount factor	γ	0.99	Policy update delay	—	2
Soft update coefficient	τ	0.005	Exploration noise std	$\sigma_{\exp}$	0.1
Replay buffer size	|D|	$10^{6}$	Target noise std	$\sigma_{\mathrm{tgt}}$	0.2
Mini-batch size	*N*	256	Noise clip	—	0.5
Actor learning rate	$\alpha_{\pi }$	$3\times 10^{-4}$			
Critic learning rate	$\alpha_{Q}$	$3\times 10^{-4}$	*Simulation/Environment*		
Temp. learning rate	$\alpha_{\alpha }$	$1\times 10^{-4}$	Episode length	*T*	200 steps
Lagrange learning rate	$\alpha_{\lambda }$	$3\times 10^{-3}$	Mean task size	$\mu_{L}$	5 Mbits
Initial temperature	$\alpha_{0}$	0.2	Computation intensity	κ	1500 cycles/bit
Initial Lagrange multi.	$\lambda_{0}$	1.0	UAV processor freq.	$f_{\mathrm{UAV}}$	2 GHz
Max Lagrange multi.	$\lambda_{\max}$	20.0	Edge server freq.	$f_{\mathrm{RSU}}$	20 GHz
Target entropy	$\bar{H}$	−dim(A)	Channel bandwidth	*B*	20 MHz
Hidden layer sizes	—	2×256	Max transmit power	$P_{\max}$	1 W
Activation function	—	ReLU	Min transmit power	$P_{\min}$	0.01 W
Warmup steps	—	5000	Mean SNR	$\bar{\Gamma }$	10 dB
			SNR std deviation	$\sigma_{\Gamma }$	5 dB
			Latency deadline	$T_{\max}$	35 ms
			Sensing duration	$T_{\mathrm{sense}}$	5 ms
			CPU capacitance coeff.	ζ	$10^{-28}$

**Table 4:** Final performance metrics (mean over last 50 epochs).

Algorithm	Violation Rate	Return	$\rho_{\eta ,\Gamma }$	Mean Latency
C-SAC	**18.9%**	−31.22	−0.894	**29.97 ms**
SAC	79.5%	−19.87	−0.736	329.49 ms
TD3-Lagrangian	41.3%	−27.13	−0.928	33.69 ms

**Table 5:** Ablation study results.

Configuration	Violation	Return	$\rho_{\eta ,\Gamma }$	Impact
Full C-SAC	18.2%	−30.48	−0.887	Baseline
*Lagrange multiplier ablations*				
Slow λ LR (0.1×)	44.4%	−26.23	−0.890	+144% violations
Fast λ LR (10×)	18.6%	−30.51	−0.892	Negligible change
Fixed λ=5	35.1%	−27.94	−0.902	Insufficient penalty
Fixed λ=15	20.4%	−29.94	−0.896	Near-optimal manual tuning
*Entropy temperature ablations*				
Fixed α=0.1	24.1%	−34.26	−0.761	Reduced adaptation
Fixed α=0.5	39.5%	−34.94	−0.601	Excessive exploration

**Table 6:** Deadline sensitivity analysis.

Deadline	C-SAC	SAC	TD3-Lagrangian
25 ms	52.4%	94.4%	58.9%
35 ms	18.3%	81.6%	31.0%
45 ms	6.9%	71.3%	22.0%
55 ms	4.6%	65.3%	16.7% *

* Anomalous: $\rho_{\eta ,\Gamma }$ drops to −0.400, indicating degraded adaptation.

**Table 7:** Channel variability sensitivity.

$\sigma_{\Gamma }$ (dB)	C-SAC Violation	TD3-Lag Violation
4 (mild)	17.1%	42.4%
8 (moderate)	18.0%	27.8%
12 (severe)	19.0%	47.9%
Range	1.9%	20.1%

**Table 8:** Lagrange multiplier bound sensitivity.

$\lambda_{\max}$	Violation	Return	Interpretation
5	35.0%	−27.96	Under-constrained
10	25.1%	−29.38	Moderate
20	18.7%	−30.51	Balanced
50	14.2%	−31.78	Conservative
100	12.4%	−34.09	Over-constrained

## Data Availability

The original contributions presented in the study are included in the article.
